# The Importance of Evaluating Positive Welfare Characteristics and Temperament in Working Therapy Dogs

**DOI:** 10.3389/fvets.2022.844252

**Published:** 2022-04-04

**Authors:** Sharmaine L. Miller, James A. Serpell, Kathryn R. Dalton, Kaitlin B. Waite, Daniel O. Morris, Laurel E. Redding, Nancy A. Dreschel, Meghan F. Davis

**Affiliations:** ^1^One Health Laboratory, Department of Environmental Health and Engineering, Johns Hopkins University Bloomberg School of Public Health, Baltimore, MD, United States; ^2^Department of Clinical Sciences & Advanced Medicine, University of Pennsylvania School of Veterinary Medicine, Philadelphia, PA, United States; ^3^Department of Clinical Studies–New Bolton Center, University of Pennsylvania School of Veterinary Medicine, Kennett Square, PA, United States; ^4^Department of Animal Science, Pennsylvania State University, University Park, PA, United States; ^5^Department of Molecular and Comparative Pathobiology and Division of Infectious Diseases, Johns Hopkins School of Medicine, Baltimore, MD, United States

**Keywords:** therapy dog welfare, positive welfare, human-dog interaction, oxytocin, cortisol, dog behavior, Animal-Assisted Intervention (AAI), Animal-Assisted Therapy (AAT)

## Abstract

To date, investigations of the welfare of therapy dogs have focused largely on examining physiological and behavioral measures that could indicate if the animal is experiencing stress or distress. However, this approach does not fully address the definition of welfare which is often described as existing on a continuum from negative (or stressful) to *positive*. With therapy dogs, it would be worth addressing if they experience positive emotional affect while working since the quality and efficacy of animal-assisted interventions for the human recipient is likely to be influenced by the animal's emotional state during the interaction. The purpose of this review is to articulate how objective measurements of the HPA axis and measurements of behavioral observations and standardized questions can be used to evaluate positive welfare in therapy dogs. A potentially relevant indicator of positive welfare is the peripheral concentration of the neurohormone oxytocin, which has been found to increase in systemic circulation within a variety of species during positive social and affiliative contexts, including during human-dog interaction. Oxytocin is also a negative-feedback regulator of the Hypothalamic-Pituitary-Adrenal (HPA) axis, which culminates with the production of the stress hormone cortisol. Cortisol is widely used as a physiological indicator to assess negative welfare states in animals, including therapy dogs. Observable behavior during interactions with humans that may convey enjoyment could provide indicators of positive welfare in dogs such as engagement in play, or human-directed affiliative behaviors including leaning against, nudging, or licking the patient. However, in assessing positive welfare, it is also critical to consider that all animal behavioral displays and physiological responses are dependent on the dog's individual (and breed) temperament. Temperament directly drives how the animal copes and responds to its current physical and social environment, including during stressful situations such as when therapy dogs interact with unfamiliar humans in novel healthcare settings. Coupled with both positive and negative physiological and behavioral welfare indicators, questionnaire data can provide further context to, and enhance interpretations of, therapy dog welfare assessment results. Overall, to date, no studies have measured all of these factors to assess therapy dog welfare.

## Introduction

Animal-Assisted Therapy (AAT) is the practice of health care professionals incorporating structured and objective-driven human-animal interaction (HAI) within patient treatment plans for the benefit of patient health (1). Animal-Assisted-Activities (AAA), another type of animal-assisted intervention (AAI), can be led by HAI professionals or volunteers, may be conducted in a variety of settings, and are meant to enhance participant quality of life, with or without consideration of health-related objectives (1). Examples include providing “motivational, educational, recreational, and/or therapeutic benefits” (1). Despite a long and rich history of examining AAI effects on human and patient welfare, investigations of potential effects on therapy dog physical health and emotional well-being, collectively termed welfare (2–4), have only begun to be extensively explored within the last two decades (5–12).

Assessment of therapy dog welfare has primarily focused on measurements of stress-related physiology, particularly the peripheral concentration of the hormone cortisol, and/or dog behaviors found to be previously associated with stressful situations (13–15), such as panting, paw-lifting, repetitive lip-licking, and avoidance behaviors (6–8, 10, 16). However, focusing on the identification of poor welfare indicators only *partially* addresses the animal welfare concept. Welfare is commonly discussed as existing on a continuum from very poor to very *good* (17–19). Therefore, consideration of positive welfare indicators *paired with* negative indicators allows for a more thorough and complete examination of a therapy dog's welfare state (16, 18). The concept of positive therapy dog welfare has only begun to be considered quite recently, with the examination of human-directed affiliative and social behaviors (10, 12, 16) and the measurement of peripheral salivary oxytocin concentration (11).

Examining whether therapy dogs experience positive affective states from interacting with patients is valuable for two reasons. First, positive human social interactions may be a core component of the welfare of domesticated animals, especially for dogs (20, 21) which have undergone thousands of years of co-evolution with, and selection by, various human populations (22–24). Second, through One Health and One Welfare perspectives, within an AAI environment, animal welfare is directly connected to recipient welfare and vice versa (25). A dog experiencing a positive affective and affiliative state during patient interaction would likely enhance the value and benefits of AAI for the human participant such as improvements in mood, reductions in self-perceived pain, and patient distress, [mood: (26, 27); pain: (28); all: (29)]. In other words, interactions with a dog that is comfortable, happy, and highly sociable are likely to be more therapeutic and enjoyable for the human recipient, than those with a dog that finds these interactions either neutral, stressful, or aversive.

An additional gap exists in the therapy dog welfare literature. An examination of animal temperament, or behavioral traits or responses that are consistently seen in similar contexts and across time (30, 31), is not commonly included within therapy dog welfare studies. Evaluation of therapy dog temperament through standardized questionnaires completed by handlers has only been employed recently (10, 16), as demonstrated in Table 1. Temperament can directly affect how an animal copes psychologically and physiologically with challenging situations or environments, including stressful ones (32), such as human-animal interactions (30). This is especially relevant to therapy dog work as its core feature is interaction with unfamiliar humans, often within novel environments (8, 10, 33). The therapy dog's temperament can therefore impact the quality of human-animal interactions with patients during AAI sessions (23).

**Table 1:** Therapy dog welfare literature examining dog temperament or human-directed dog affiliative behaviors.

**Reference**	**Brief purpose of study**	**How was dog temperament or positive welfare indicators assessed?**
Piva et al., (12)	To assess the welfare of a recently adopted therapy dog within a facility for elderly Alzheimer's patients.	Researchers collected questionnaire data from nursing staff on, e.g., the therapy dog's playfulness and sociability towards patients at the beginning, middle, and end of a series of 9 AAA sessions.
McCullough et al. (10)	To assess the welfare of a therapy dog interacting with pediatric oncology patients.	Human-directed dog affiliative behaviors were measured by coding the frequency of these behaviors listed within a descriptive ethogram. Affiliative behaviors included the number of times dogs leaned or rested against the patient or object, licked the patient, paw-lifted towards the patient, engaged in a play-bow (indicating wish to play), etc. Handlers completed the C-BARQ questionnaire online and were asked how their dogs responded to different stimuli and daily-life events within their home environment, such as when interacting with strangers.
Corsetti et al. (16)	To assess the behavioral displays of dogs while participating in AAT sessions with patients suffering from mental illness or “psychomotor” ailments.	Researchers used a diverse ethogram that included examining affiliative behaviors. Affiliative and playful behaviors included wagging tail, inviting play by engaging in a play-bow, and licking the patient's hand. Researchers measured these behaviors if the dog interacted with any human present before, during, and after AAT, including the patient, behavioral observer, or study operator. Researchers also assessed dog temperament by having dog handlers complete the C-BARQ questionnaire.

Assessing temperament can provide further information on how individual therapy dogs regularly behave and can clarify drivers of any unusual behaviors observed during therapy sessions. Specifically, collecting and considering information on how the dog typically behaves in similar situations outside of work, in their normal environment, may improve our ability to predict and understand how the dog will interact with human recipients during AAI. Furthermore, although there appears to be common use of temperament or behavioral examinations to select dogs fit for AAI work (34), especially those with a calm temperament, the lack of standardization and longitudinal follow-up makes it difficult to determine instrument validity (23, 35). Validated pet and working dog temperament questionnaires may be useful to monitor therapy dog welfare and evaluate long-term success of identifying dogs suitable for AAI work. Collecting information on the therapy dog's background, e.g., past experiences, and demographics, e.g., age, can also aid in further clarifying temperament results as these characteristics can affect temperament trait outcomes.

The goal of this review is to enhance the assessment instrumentation of animal welfare professionals who aim to assess and improve therapy dog welfare. This review provides information on the background and measurement of potential positive-welfare measures, such as human-directed social and affiliative behaviors and peripheral oxytocin concentration within dogs. Examples of dog behaviors potentially indicative of a positive welfare state are provided within a context of recent examinations of human-directed social behaviors among therapy dogs, as shown in Table 1 (10, 16), and other working or pet dogs from the more abundant human-dog interaction literature. Given that prior studies have identified heightened peripheral oxytocin to be associated with dogs experiencing positive affiliative encounters with humans (see Table 2), the utility of measuring peripheral oxytocin within a therapy dog welfare assessment is also discussed. This review closes with a discussion on the methods and potential value of utilizing a therapy dog temperament assessment, including examining traits directly relevant to positive HAI encounters such as sociability and playfulness and its usefulness for AAI dog selection.

**Table 2:** Examples of studies examining salivary peripheral oxytocin concentration in pet or working dogs during human-animal interaction.

**Reference**	**Brief purpose of study**	**Biomarker(s) used**	**Methods (type of social interaction, oxytocin sampling)**
MacLean et al. (36)	To examine oxytocin levels of assistance dogs before and after dogs interacted with the experimenter.	Serum (*via* blood sampling) and salivary oxytocin	Pilot study: serum and salivary samples were taken immediately before HAI, 5 min after the start of HAI, and 10 minutes after HAI. HAI consisted of the researcher petting the dog, speaking in a friendly quiet voice, and engaging in eye contact with the dog. Experiment 1: Half of sample experienced HAI and half did not (control group). Blood and salivary samples were collected before and 10 minutes after HAI. Dogs were allowed to initiate all HAI. Saliva samples were collected with a Salimetrics children swab. A Cayman Chemical Enzyme-Linked Immuno assay (ELISA) kit was used to assess peripheral oxytocin concentration.
Clark et al. (11)	To examine the welfare of therapy dogs during AAA with Chronic Fatigue and Fibromyalgia patients within an outpatient setting.	Salivary oxytocin	Dog salivary oxytocin levels were collected with a swab (2 min max) before and after a 20-minute AAA session. HAI was not standardized to not compromise quality of therapeutic session. Samples were processed *via* liquid chromatography-mass spectrometry.
Ogi et al. (37)	To examine the effects of positive human-dog interaction, and an acute stressor on guide dogs in training.	Salivary oxytocin and Salivary cortisol	Dogs were exposed to two different conditions one week apart consisting of being exposed to an unfamiliar room where they experienced a positive interaction with study researchers for 5 min. Researchers stroked and spoke calmly to the dogs. The second study condition involved dogs experiencing an unfamiliar room while being socially isolated for 5 min (acute stressor). Saliva samples were collected with salivettes (Sarstedt Company) before, after, and 15 min post each condition. Samples were analyzed with a Cayman Chemical ELISA kit.
Lopez-Arjona et al. (38)	To test the validity of different hormone detection and extraction methods of peripheral oxytocin within pet dogs.	Salivary oxytocin	Within the dog's home environment, saliva samples were taken before, immediately after, and an additional 15 minutes after dogs experienced an affiliative interaction with their owners. The interaction consisted of owners stroking their dogs and speaking softly to them. Saliva samples were collected with a sponge placed within the dog's month for 1 minute, and then placed within Salivette tubes (Sarstedt). Samples were processed *via* a monoclonal AlphaLISA, polyclonal AlphaLISA, or standard ELISA kit, for comparison. Some samples also experienced reduction/alkylation extraction for further comparison.

## Animal Welfare and the Emergence and Measurement of Positive Welfare

Animal welfare has been defined as the physical and emotional state of an animal in relation to how it copes with its current environment (2–4, 39). When an animal is unable to cope with highly challenging or aversive stimuli within its environment, i.e., external (behavior) and internal physiological stress coping responses are ineffective, this could cause the animal to enter a state of distress, and thus a poor welfare state (2, 4, 40, 41). Over time, repeated or continued experience of distress, can lead to damaged and dysfunctional physiological functioning, which can promote poor physical and mental health outcomes. Therefore, protecting animals from stressful or distressing experiences has been the fundamental purpose of assessing the welfare of all animals living under various environmental conditions (42, 43).

It is evident that this mindset has also been widespread within past examinations of therapy dog welfare [e.g., (5–9, 44–46)]. It is important to understand that achieving good or positive welfare does not solely constitute protecting an animal from negative experiences (41), but also involves providing environmental conditions in which an animal may encounter positive experiences (47).

The discussion of the positive welfare concept encompasses four key characteristics, which are: quality of life, happiness, positive emotions, and positive affective engagement (48). All these dimensions can be applied to therapy dog welfare. Quality of life pertains to the frequency and degree of experiences an animal encounters within its life, i.e., an animal that encounters more positive experiences is said to have a better quality of life than an animal that has accrued more negative experiences (48). The purpose behind investigating a therapy dog's welfare state is inherently meant to identify risks in the therapy dog's quality of life, specifically pertaining to work. For example, therapy dogs that experience a high number of work sessions in a given period of time (5) or primarily work on-leash opposed to off (6) may experience heightened stress, and as a result, a worse quality of life.

In contrast to quality of life, happiness can be thought of as a singular psychological or emotional state where the animal has obtained some facet of positive welfare (48). The concepts of positive emotions and positive affective engagement can allow for understanding in how this can be achievable in therapy dogs. “Positive emotions” involve the acceptance that animals can experience both negative *and* positive emotions or feelings (18, 41, 48, 49). An animal's emotions or feelings are dependent on various factors including motivational state or “wants” and “likes,” past life experiences, temperament (41), and past and current environment. Within the context of therapy dog welfare, it is crucial to consider all these components, as all of them can affect the animal's present emotional state while working.

One way to promote positive emotions in animals is by providing opportunities for positive affective engagement, or positive HAI encounters (21, 41, 48, 50). Within therapy dog work, therapy dog handlers and AAI personnel could promote positive welfare by encouraging play between dogs and patients, which may in turn encourage patient-directed affiliative behaviors within dogs. To identify these behaviors, handlers should familiarize themselves with dog-human social communication to be able to interpret what the dog is trying to communicate, both to them and to the AAI participant or patient (35, 51). While these same social displays can be observed in wolves during intraspecific interactions, they appear to have been amplified in the dog by thousands of years of selection for interspecific communication with humans (22, 52, 53). Due to this extensive co-evolutionary history, human-directed social behaviors in dogs are critical and frequently used elements of their behavioral repertoire and will likely be repeatedly present during HAI situations.

In short, dogs communicate directly with their human partners through behavioral displays by changing their body language, such as their facial expressions (e.g., moving their ears, or changing eye shape), and their overall body position and posture (52, 54). The value of understanding and including behavioral measurements within a therapy dog welfare assessment is that they are directly observable in real-time, compared to equally valuable physiological indicators where there is a lag between sample collection (e.g., saliva or blood) and reporting of laboratory results. Therefore, behavioral measurement can be a first-line of identification if the dog is enjoying the session or is experiencing distress. Although recent therapy dog welfare studies have not always included behavioral measures [e.g., (11, 46, 55)], we recommend that they be included in future studies to better evaluate and monitor the welfare of therapy dogs while they work.

### Animal Behavior as a Potential Indicator of Positive Welfare

Play and affiliative behaviors may be associated with positive welfare in dogs. Dogs solicit play by displaying a characteristic “play bow,” in which the dog lies down on its front legs while elevating its rear end (52, 54). To solicit affiliative attention, dogs wag their tails in a loose or relaxed manner (52), rub their bodies or heads against a human recipient, or engage in social licking (56), all of which may foster social bonding or help reduce social tension (41). Two recent therapy dog welfare studies have included play and human-directed affiliative behavioral measures centered around human-dog social interaction.

As shown in Table 1, behaviors examined included the frequency and/or duration of dogs lifting a relaxed foreleg or paw towards the patient to garner attention, displaying play-bows or rolling over, and engaging in direct social contact with the patient such as licking, nudging, and resting their body against them (10, 16). Direct measures of HAI within an assessment of therapy dog welfare may especially be critical as direct physical contact with the patient is likely a strong mechanism behind positive health effects of therapeutic sessions for patients, not just the presence of the animal itself (21).

Play is frequently observed under low-threat conditions, across a variety of different species (57, 58). Despite potential fitness costs, conducting play behavior may indicate that the animal feels comfortable or relaxed within its environment. Secondly, human-directed play [e.g., (59, 60)] and affiliative behaviors in dogs [e.g., (10)] may incur specific health benefits, i.e., reducing maladaptive stress responses when experiencing non-threatening conditions. For example, Rossi et al., (60) found that a reduction of peripheral cortisol was associated with an increase in the frequency of play behaviors in pet dogs, e.g., play bow, paw lift, soliciting chase (60). In Hungarian border guard dogs, salivary cortisol decreased after dogs played with their handlers (*p* = 0.05), i.e., tugging a toy and retrieving the object after being thrown (59).

Similarly in therapy dogs, *via* a linear regression analysis, McCullough et al., (10) found that an increase in salivary cortisol concentration was associated with a decrease in the frequency of affiliative behaviors (*p* = 0.005), thus a decrease in cortisol concentration was also associated with a higher frequency of affiliative behaviors (10). It is possible that the minimization of stress responsivity may have promoted an increase in dog affiliative displays towards patients (10) and play behaviors towards experimenters (60), or vice versa. Both studies highlight how play and affiliative behaviors may provide a useful indicator to determine if therapy dogs are experiencing or approaching a positive welfare state.

In addition to play and affiliative behaviors, measuring therapy dog activity may provide further information on the dog's current welfare state. For example, a therapy dog that is consistently engaging with its physical and social environment may feel comfortable, motivated to participate in human interaction, and overall be experiencing a positive emotional state. Whereas a therapy dog that is avoidant or unresponsive to human interaction may be uncomfortable, fatigued, or experiencing distress. Previous therapy dog welfare studies have measured activity *via* the frequency or duration of therapy dogs spending time resting, exploring (e.g., sniffing environment), standing, sitting, or walking (7, 8). It is important to emphasize that multiple categories of relevant behavioral measures (e.g., play or affiliative, activity, stress-associated), as well as physiological indices should be assessed to provide a clear picture of the context and potential motivation behind behaviors performed during work sessions.

## The Physiological Importance of Peripheral Cortisol and its Relationship with the Neurohormone Oxytocin

**Figure 1:**
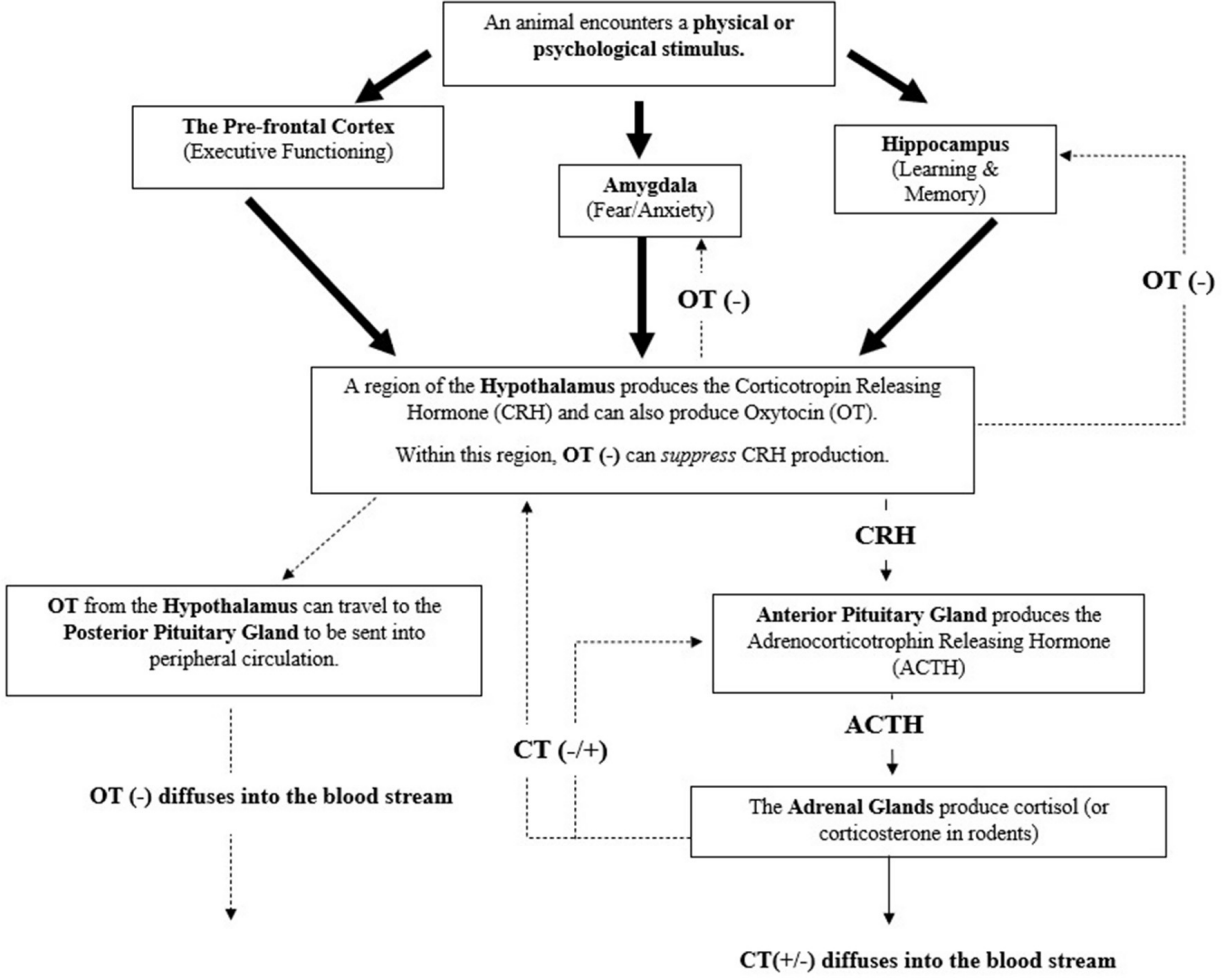
The mammalian hypothalamic pituitary-adrenal axis (HPA) and a summary of its complex relationship with the hormones Cortisol (CT) and Oxytocin (OT) (61–64) Negative feedback (represented by minus signs) can be initiated by oxytocins to lesson cortisol's production. Cortisol can act as a negative or positive feedback mediator (represented by plus sign) to further lesson or amplify its production, respectively. Large arrows represent sensory signals that are integrated to initiate the HPA axis.

In studies of therapy dog welfare, measuring peripheral cortisol has commonly been used as a key welfare indicator to investigate the extent of internal stress coping responses [e.g., (6–11)]. The mammalian Hypothalamic-Pituitary Adrenal Axis (HPA Axis), shown in Figure 1, is responsible for the production of glucocorticoids, or the hormone cortisol (corticosterone in rodents) (61–63, 65–67). HPA axis activation can be a critical adaptation for an animal to successfully cope with stressful circumstances. Cortisol mobilizes and mediates internal coping responses by initiating functional changes within various physiological processes and pathways across different brain and body systems [(65, 66, 68) as cited in (40, 62, 63)]. These physiological modifications in turn promote or are promoted by external stress coping responses, i.e., changes in animal behavior, and all these changes allow an animal to respond more efficiently to the heightened energy demands that come with navigating challenging situations (69, 70). For example, McCullough et al., (10) found when statistically controlling for affiliative behaviors, higher salivary cortisol concentration per session was associated with an increase in stress-associated behaviors in therapy dogs, e.g., body shaking or trembling, lip-licking with no food stimuli present [*p* = 0.039; (10)]. These results match expectations that heightened physiological stress responsivity (e.g., cortisol production) would co-occur with more frequent displays of stress-associated behaviors, indicating that the dog is engaging external and internal stress coping responses.

When stress-associated brain-body pathways, such as the HPA axis or the Sympathetic-Adrenal-Medullary axis (SAM), principally involved with acute stress and the *fight* or *flight* response, become frequently activated or long-lasting, this can lead to dysregulation and dysfunction of stress response-related pathways and in turn result in bodily or neurological damage (40, 70, 71). Measuring cortisol has therefore become a potential means to identify if an animal is experiencing or approaching a poor welfare state.

To mitigate this, particularly when responsivity to a stressor(s) is no longer warranted, the HPA Axis has several negative feedback mechanisms to promote movement back to a baseline, homeostatic state (61, 64, 72–74). For example, cortisol can act upon glucocorticoid receptors within the hypothalamus or amygdala (principally involved in mobilizing fear or anxiety) to lessen the production of the corticotropin-releasing hormone (CRH) or act upon the anterior pituitary gland to lessen production of the adrenocorticotropic hormone (ACTH) (61–63). Cortisol can also act upon the hippocampus, a region of the brain principally involved in learning and memory, to produce another neurohormone to stunt production of CRH within the hypothalamus (61, 63, 64). It is important to note that cortisol can also heighten its own production by acting upon these same regions or certain areas of the prefrontal cortex (64), which are responsible for executive functioning.

In addition to cortisol (and corticosterone), the hormone oxytocin is also involved with the negative feedback inhibition of the HPA axis (74), as illustrated in Figure 1. As with CRH, oxytocin is also produced within the hypothalamus. Within this region oxytocin can initiate the inhibition of CRH production, preventing CRH's ability to initiate the HPA response by acting upon the anterior pituitary gland (75). Oxytocin can also move to other midbrain structures, such as the hippocampus or amygdala to signal the inhibition of cortisol production and to the posterior pituitary gland for storage or release into peripheral circulation (22, 73, 74). When oxytocin is released into peripheral circulation it can induce downstream inhibitory effects on the HPA (and SAM) axis, such as reducing heart and respiratory rate (22, 73, 74). Oxytocin's involvement with the regulation of the brain and body's response to stress highlights its relevance in adding it as a physiological measure, in conjunction with cortisol, within an assessment of therapy dog welfare. Furthermore, oxytocin's role in attenuating internal stress coping strategies highlights its potential in indicating if an animal is approaching a positive welfare state.

Although the hormone oxytocin is primarily known for being a key player in physiological and behavioral functions associated with reproduction, oxytocin has also been found to be associated with promotion of affiliative social behavior and social bonding across a variety of different contexts and species [e.g., (22, 76, 77)]. For example, Montane voles (*Microtus montanus*) that gave birth were found to have heightened oxytocin affinity (within the amygdala) and also engaged in affiliative behavior with non-familial pups more, compared to virgin voles (78). Chimpanzees that groomed bonded social partners were found to have higher levels of urinary oxytocin compared to when grooming non-bonded partners when or experiencing no social interaction (79). In humans, mothers and fathers who engaged in more affiliative contact with their infant(s) had a higher concentration of salivary oxytocin compared to before infant interaction (80, 81).

Due to oxytocin's strong association with facilitating affiliative bonds and modulating social behavior, oxytocin responsivity has been repeatedly examined in the context of human-dog interaction [Reviews: (20, 22, 82)]. Measuring peripheral oxytocin concentration in dogs has been proposed to be a potential means of assessing positive emotional affect during HAI (20), including within therapy dogs while they interact with AAI participants (11).

## Human-Dog Interaction and Measuring Endogenous Peripheral Oxytocin: The Benefits and Caveats of Blood, Urine, and Saliva Sampling

A minority of studies have measured peripheral oxytocin in the context of therapy dog-patient interaction, such as *via* salivary sampling (Table 2). Within the broader human-dog interaction literature, a larger number of studies have examined peripheral oxytocin's relationship with human-dog interaction, particularly within an affiliative context. In this literature, there is substantial heterogeneity in sample collection methods to ascertain peripheral oxytocin concentration within dogs (37), with blood sampling previously appearing to be the most frequently used [e.g., (83–87)] followed by urine sampling [e.g., (88–91)]. Recently, saliva sampling has emerged as a leading method [e.g., (36–38)].

Within dogs, studies examining peripheral oxytocin's relationship with positive human-dog interaction seem to be split into two domains. The first, exogenous studies, measure peripheral oxytocin after intranasal or intravenous administration [e.g., (88, 92)], and the other, endogenous studies, measure peripheral oxytocin without such administration [e.g., (36, 84, 85); Reviews: (20, 22, 82)]. For this review, exogenous studies were excluded because of several confounding factors that could potentially affect the output of peripheral oxytocin. For example, exogenous study results may depend on original individual endogenous oxytocin levels (20, 92), and high doses of exogenous oxytocin administered can result in neurological over-saturation of oxytocin hormone receptors, causing oxytocin to bind to vasopressin (AVP) receptors (AVP being a feedback promoter of the stress response) [(93) cited in (20)]. Finally, the dosage of oxytocin administered is not standardized across studies and greatly varies (20). These reasons can make it difficult to compare exogenous study results to those from endogenous studies.

With endogenous studies, specifically with blood sampling, several studies have detected statistically significant increases in dog serum oxytocin concentration after dogs experienced positive HAI [e.g., (36, 83–85)], including after dogs interacted with their owners (83, 85), after dogs interacted with owners or non-owners (84), and after dogs reunited with their owners following a period of separation (86). Still, caveats exist with blood sampling as the procedure itself (94) and maintenance of venous access is invasive and can induce stress (36, 85). Some studies have tried to circumvent this by using in-dwelling intravenous catheters to allow for quicker and less disruptive sample collection (e.g., (84, 85)]. Despite this procedure and having a relaxation period following insertion [e.g., 10 min: (84); 30 min: (85, 87); 60 min: (86)], in-dwelling catheters may still alter physiological and behavioral results as they are not normally present during baseline conditions for the dogs.

To measure peripheral oxytocin concentration with urine sampling, several issues can prevent accurate sampling, such as the potential to capture wide windows of hormone activity, potentially due to the urinary system not being fully flushed before an experiment begins (36, 89, 95). Urine samples also need to be and are often collected 30–90 min after exposure to a stimulus (88, 90, 91, 96), which can complicate the interpretation of the *immediate* effect of human-dog interaction (36). Despite these difficulties, some prior studies have identified increases in dog urinary oxytocin after they experienced human affiliative attention, such as after dogs have experienced extended eye-gazing (88) and physical contact (91) from their owners. In contrast, other studies have found no significant differences in urinary oxytocin concentration between baseline conditions and 30 minutes after dogs interacted with their owners (90), and 60 min after dogs interacted with owners (89). Thus, variation in study findings have generated concerns about the reliability of urine sampling.

Recently within the human-dog interaction literature, salivary oxytocin has emerged as a potential promising new biomarker for non-invasive measurement and detection of changes in dog peripheral oxytocin concentration [e.g., (36–38)]. A summary of study design and salivary sampling methods is provided within Table 2. It was found that dogs exposed to HAI had significantly higher salivary oxytocin after 10 min of continuous human interaction compared to a control group which did not experience HAI [*p* = 0.02; (36)]. Dogs that experienced HAI also had a significant increase in salivary oxytocin compared to baseline [*p* < 0.01; (36)]. Increases in salivary oxytocin concentration were also found to be positively associated with, and predicted by, the extent of dog affiliative behavior displays, with behavior scores indexed by a principal components analysis (*p* = 0.04). Within this study and in efforts to validate the measurement of oxytocin responsivity within dog saliva, serum oxytocin concentration was also examined. Although there were no significant differences between the HAI and control group, within the HAI group, there was a significant increase in serum oxytocin after dogs experienced HAI compared to before interaction [*p* = 0.05; (36)].

The concentration of serum and salivary oxytocin has been compared before and after dogs nursed their litters to further test the validity of a salivary oxytocin biomarker within an affiliative context (95). Within a parent-offspring context, similar increases in serum and salivary oxytocin after nursing were detected, where serum oxytocin increased at an average of 46.4% and salivary oxytocin at 69.3% (95). Only the change in salivary oxytocin was significant (*p* < 0.01), whereas a trend was detected for an increase in serum oxytocin [*p* = 0.07; (95)]. Overall, both studies provide compelling evidence that, like blood sampling, saliva sampling can capture relatively rapid changes in oxytocin responsivity within an affiliative context.

In another study, dogs that showed minimal signs of stress during saliva collection showed statistically significant increases in oxytocin concentration both immediately (*p* = 0.0041) and 15 min after interacting with their owners in their home environment [*p* = 0.0079; (38)]. Within the context of working dogs, increases in salivary oxytocin were also found after guide dogs in-training interacted with their handlers [*p* = 0.036; (37)]. It was also found that when exposed to a control condition or novel stressor, i.e., the absence of human interaction, there were no differences in salivary oxytocin concentration (37).

MacLean et al., (36) and Ogi et al., (37) had uniform study samples predominantly consisting of one dog breed and found significant results (Table 2). In contrast, Clark et al. (11) and Lopez-Arjona et al. (38) both examined the peripheral oxytocin reactivity of various breeds and found conflicting results. In Clark et al. (11), no change was detected in salivary oxytocin concentration after therapy dogs interacted with patients [*p* = 0.85; (11)]. It is possible that therapy dogs in Clark et al., (11) did not garner an affiliative reaction during patient interaction. Differences in peripheral oxytocin reactivity may also exist between dog breeds, which would be expected to increase the variability of results and could weaken significance.

In Lopez-Arjona et al., (38), a significant difference in dog salivary oxytocin concentration after HAI was only detected when data were separated by reactivity to sample collection. Dogs that were less reactive to sample collection, e.g., fewer changes in position or attempts to remove the collection swab, showed an increase in salivary oxytocin concentration after interacting with their owner, whereas there was no change for the higher reactivity group (38). Stratifying results based on reaction to fearful stimuli [e.g., sample collection: (38)] may have also coincidentally separated dogs based on their existing temperament.

## Dog Breeds and Differences in Temperament

Common temperament traits identified within dogs are excitability, aggressiveness, curiosity/fearlessness, trainability, sociability, playfulness, and a higher-order trait known as boldness [propensity to take risks; (30)] which can influence the expression of all these traits (31, 53, 97–102). These traits have been identified by exposing dogs to a series of different testing situations and measuring their behavioral responses based on an ethogram (97, 99) or through standardized ratings by expert behavioral and veterinary clinicians (100). Questionnaires have also aided in the identification of temperament traits within dogs by owners providing information on how their dog(s) typically behave across a variety of situations (103, 104). Consistency over time and testing situations are the two criteria that define temperament (30, 31, 53); temperament traits such as playfulness, curiosity/fearlessness, sociability, and aggressiveness have been shown to be consistent over time within dogs and across similar testing situations (99).

With dogs, it is widely accepted that behavior differs between breeds, with empirical evidence to support this [e.g., (105–107)]. For example, through secondary analysis of existing data from the Swedish Dog Mentality Assessment (DMA), an assessment composed of various test batteries, Svartberg (105) found that Golden Retrievers ranked relatively low in aggression (#22/31), low in curiosity/fearlessness (#26/31), and relatively high in sociability and playfulness (#5 and 12 respectively) compared to other breeds tested such as the Belgian Malinois, Australian Shepherd, American Staffordshire Terrier, Parson Russell Terrier, or Great Swiss Mountain Dog (all of which ranked in the top 5 for aggression).

Within the context of therapy dog work, McCullough et al. (10) examined dog temperament by using the Canine Behavioral Assessment and Research Questionnaire (C-BARQ) and found that dogs prone to *stranger-directed fear* were not as affectionate towards patients, compared to dogs less fearful of strangers (Table 1). McCullough et al. (10) found Labrador and Golden Retrievers were more affectionate than other breeds (e.g., mixed breeds, Newfoundlands, a Border Collie mix) and displayed more human-directed affiliative behaviors during therapy sessions (10). Similarly, Serpell and Duffy (107) found that Golden and Labrador Retrievers were less fearful towards strangers and less sensitive to touch (e.g., petting), compared to the overall average of the sample population consisting of various dog breeds. All these results point to the potential value of particular breeds as therapy dogs, given that these dogs are required to interact with unfamiliar individuals *via* physical interaction.

However, Svartberg (105) found Labrador Retrievers ranked higher in aggression (#16/31) than Golden Retrievers despite ranking higher in sociability and playfulness (#3 and 6 respectively) compared to Golden Retrievers and other breeds examined. Differences in temperament have also been identified at the individual level with a sample of Golden Retrievers (16). However, it is important to note that due to a small sample size (n=9) there is likely low statistical power with results obtained.

Conflicting results of the studies mentioned above point to another, more critical component that needs to be considered when assessing dog temperament. Temperament can vary between populations (such as dog breeds), but it most certainly always varies within a population, specifically at the individual level (30, 31, 33, 108). Within a population, expression of a trait varies along a continuum, with typically most of the population lying within the middle of a distribution (30, 31, 108). For example, for the trait boldness, individuals vary from being very bold to very shy with these two representing extremes at each end of this continuum. Overall, it is critical to assess and consider *individual* therapy dog temperament, as temperament can be highly variable from one individual to the next and assumptions on temperament simply based on breed may not apply reliably (33, 35).

### Factors That Can Affect Temperament and the Importance of Re-evaluation for AAI Dog Selection

Many factors can and do impact an individual dog's temperament such as rearing and current physical and social environment, and demographic characteristics such as age, size, sex, and reproductive status i.e., altered, or unaltered [e.g., (103, 106, 109)]. For example, military working dogs in training that were left alone for long periods of time during early-life scored higher for C-BARQ questionnaire items related to *trainability, hyper*-*activeness* and *chase-proneness* compared to dogs that were not left alone as long (109). Through the Monash Canine Personality Questionnaire (MCPQ) (103), it was found that increased time spent inside was correlated with low boldness scores among dogs (*p* = 0.007). Starling et al., (106) and Ley et al., (103) found older dogs were less bold than younger dogs *via* a modified C-BARQ questionnaire (*p* < 0.001) and MCPQ (*p* < 0.001), respectively. Starling et al., (106) also found male dogs were bolder than females and dogs that were not spayed or neutered were bolder than dogs that were altered (*p* < 0.001 and *p* = 0.023), respectively, when all breeds were included in analysis (106). When not considering breed in analysis, it was found that smaller dogs were less bold than extra-large dogs [*p* < 0.001; (106)].

Considering the above factors in conjunction with individual dog temperament may also be important for AAI dog selection. Within the US, re-evaluation of therapy dog temperament appears to not be consistently conducted, according to a randomized sample of AAI organizations (34). However, the results of Ley et al., (103) and Starling et al., (106) suggest that therapy dog behavior may change as the animal ages or if it is sterilized. It is also important to consider that an individual therapy dog is constantly facing new experiences both at work and at home, all of which could result in long-term changes in behavior, especially if these experiences are aversive. It is also possible that prolonged absence from therapy dog work, e.g., for medical treatment of the dog or because of program-related factors such as COVID-19 suspension of activities, could affect current and future behavioral responses, although this has yet to be evaluated explicitly. For these reasons it is critical that the background and temperament of the therapy dog be regularly evaluated over time, to continue assessing if they are still fit to work within an AAI environment. An additional benefit of periodic re-evaluation is that it allows for the consistent collection of the most up-to-date information relevant to the therapy dog's current physical and emotional state.

### Measuring Dog Temperament and Including Sociability and Playfulness

A number of reliable questionnaire instruments have been developed to assess aspects of temperament or personality in dogs, including the Positive and Negative Affect Scale or PANAS (110), the Monash Canine Personality Questionnaire (103), and the C-BARQ (98, 109, 111–113). Because the C-BARQ was originally designed to assess pet behavior problems, such as fear or aggression towards strangers (98, 114), including additional validated questionnaire items related to sociability and playfulness (101, 102) would be of value to determine if therapy dogs are experiencing positive welfare while working. Furthermore, playfulness and sociability towards others are often key behavioral traits that are desired within therapy dogs (35).

Svartberg (102) deployed a modified C-BARQ with additional sociability and play questionnaire items for handlers with dogs that completed the Dog Mentality Assessment (DMA; created by the Swedish Working Dog Association). *Via* Principal Component Analysis, three factors were identified among questionnaire items, with high internal consistency and individual high factor loadings (102). Factors identified were *stranger-directed interest*, e.g., significant questionnaire items included “enjoys being petted by strangers,” *play behavior with human counterparts*, e.g., “retrieves play objects and initiates play,” and *dog-directed interest* (102). The first two factors are the most relevant to therapy dog work as dogs often interact with humans and not with other therapy dogs during work sessions. There were statistically significant correlations between dog trait scores from DMA performance and sociability and playfulness-related factor results, providing evidence for the reliability of these measures. Specifically, dog sociability trait scoring from the DMA was positively correlated with *stranger-directed interest* scoring (*p* < 0.05), and dog playfulness DMA scoring was positively correlated with *human-directed play interest* scoring [*p* < 0.05; (102)].

Similar results were also detected with the Behavior and Personality Assessment (developed by the Swedish Kennel Club) to create a more inclusive test-battery assessment for breeds of all sizes, compared to the DMA (101). Within the study sample, it was found that the factors *stranger-directed interest* and *human-directed play interest* again showed high internal consistency, and each factor was correlated with different assessment trait scores related to human-directed sociability and playfulness (101). The results of Svartberg (102) and Svartberg (101) suggest that dog behavior during sociability-related test batteries were correlated with the trait scoring results for modified C-BARQ questionnaire items pertaining to play and sociability. Validated questionnaire items that captured *stranger-directed interest* and play behavior could be used successfully in a modified C-BARQ to assess these traits within therapy dogs and consequently add a temperament measure relevant to the positive welfare concept.

## Conclusion

To create a more thorough examination of a therapy dog's welfare state, assessments should include both negative *and* positive physiological and behavioral indicators. Positive therapy dog welfare is critical to assess because the experience of good welfare is not solely the absence of stressful or aversive experiences. Rather, focus should be placed on assessing if the dog is comfortable and experiencing enjoyment while working. This can be achieved by examining behavioral displays relevant to positive emotional affect such as human-directed affiliative or play behaviors that promote human-dog interaction. Measuring peripheral oxytocin levels within dogs may also be of interest as this hormone has been found to be associated with positive human-dog interactions in past research. Furthermore, the consideration of individual dog temperament is critical to provide further context on the therapy dog's typical behavior outside of work sessions which can aid in the interpretation of the dog's welfare state while working.

The proposed methodology may also be valuable in identifying and selecting dogs best suited for AAI work. For example, therapy dogs that consistently display high *human-directed play interest*, a higher frequency of affiliative behaviors than stress-associated behaviors, and attenuated cortisol and heightened oxytocin over time may have a lower risk of experiencing a poor welfare state compared to dogs with the opposite behavioral and physiological profile. Dogs with the former profile may also enjoy AAI more, be at a greater odds of experiencing a positive welfare state, and therefore could enhance the quality of AAI sessions and promote beneficial effects for recipients.

## Author Contributions

SM, JS, ND, and MD: review conception. SM: manuscript drafting. JS, KD, KW, DM, LR, ND, and MD: manuscript revision. All authors contributed to the article and approved the submitted version.

## Funding

Funding for SM, JS, KD, KW, DM, LR, and MD was provided by a grant from the NIH Eunice Kennedy Shriver National Institute of Child Health & Development (Grant #: R01HD097692). MD additionally was supported by the NIH Office of the Director (Grant # K01OD019918).

## Conflict of Interest

The authors declare that the research was conducted in the absence of any commercial or financial relationships that could be construed as a potential conflict of interest.

## Publisher's Note

All claims expressed in this article are solely those of the authors and do not necessarily represent those of their affiliated organizations, or those of the publisher, the editors and the reviewers. Any product that may be evaluated in this article, or claim that may be made by its manufacturer, is not guaranteed or endorsed by the publisher.
