# Supplementary material for: The Importance of Evaluating Positive Welfare Characteristics and Temperament in Working Therapy Dogs
Source: Front Vet Sci. 2022 Apr 4;9:844252. doi: 10.3389/fvets.2022.844252 (PMC9014261; doi:10.3389/fvets.2022.844252)
Supplement: Supplementary file 1 [file Data_Sheet_1.pdf]

The importance of evaluating positive welfare characteristics and temperament in working therapy dogs

Supplementary Material

Literature Review Methods

Literature discussed within this review were captured through multiple searches with flexible and relevant terminology for each topic discussed. Specifically, literature pertaining to animal welfare and positive welfare history and methodology, oxytocin physiology pertinent to stress response physiology, oxytocin's relationship with dog social behavior during human-animal interaction, oxytocin sampling methodology, and working dog temperament and its assessment, were searched for using the following databases: Scopus, PubMed, Science Direct, and Agricola. The last search was completed on October 26th, 2021.

Literature searches were completed by one of the authors (SLM) and the search strategies and formation of this review was framed with a narrative review approach. In addition, for feasibility purposes the studies discussed are all examples of existing literature pertaining to these topics and do not constitute the entire full body of literature for every topic discussed. The likelihood that empirical studies or literature reviews were missed is possible, despite the first author sifting through hundreds of reviews and empirical studies related to the topics discussed.

For this review several search terms were utilized across the mentioned research literature databases and searches were limited to title, abstract, and keywords only. The same search terms were utilized across all databases and different search terms were used for each topic discussed within this review.

Several different terms for therapy dog work exist and thus several search terms were used to identify therapy dog welfare studies, which were: "animal-assisted-therapy AND dog welfare," "animal-assisted-activity AND dog welfare," "animal-assisted-intervention AND dog welfare." Therapy dog studies conducted in healthcare settings including hospitals (McCullough et al., 2018), nursing homes (e.g., Piva et al., 2008), or outpatient facilities (e.g., Corsetti et al., 2019) were targeted to promote benefits for future AAI patient research.

For animal welfare related topics, the search terms "animal-welfare-science," "animal-welfare AND assessment or measurement," "companion-animal-welfare," "animal-welfare AND stress coping," "positive-welfare," and "positive-animal-welfare" were used.

For stress coping and physiology research the search terms “stress physiology OR hypothalamic-pituitary-adrenal axis,” and “animal-welfare AND stress coping” were used. For peripheral oxytocin physiology, human-animal-interaction, and dog social behavior related topics, the search terms “human-dog social and play behavior,” “oxytocin AND social-bonding AND social-behavior,” “human dog social interaction,” “oxytocin AND stress physiology OR hypothalamic-pituitary-adrenal axis,” “oxytocin physiology AND human dog interaction,” were used.

For topics related to animal and dog temperament the search terms “dog temperament OR personality,” “animal temperament OR personality,” “working dog temperament OR personality,” “working dog OR therapy-dog AND temperament OR personality,” and “working dog temperament OR personality questionnaire” were used. Temperament and personality were both used during searches to better capture literature discussing consistent behavioral patterns across time and similar contexts, in dogs. Although temperament and personality are commonly referred interchangeably (e.g., Réale et al., 2007) than parsed apart, the former was chosen to avoid any potential for anthropomorphism. Lastly, if literature relevant to a topic was found during a search for another topic, the article was still selected and included within this review to promote capturing of relevant content for this review.
